# Advances in DLL3-targeted therapies for small cell lung cancer: challenges, opportunities, and future directions

**DOI:** 10.3389/fonc.2024.1504139

**Published:** 2024-12-05

**Authors:** Jianhua Ding, Chaihong Yeong

**Affiliations:** Taylor’s University, Subang Jaya, Selangor, Malaysia

**Keywords:** small cell lung cancer, delta-like ligand 3, targeted therapy, antibody-drug conjugates, bispecific T-cell engagers

## Abstract

Small cell lung cancer (SCLC) remains one of the most aggressive and challenging malignancies to treat, with limited therapeutic options and poor outcomes. Recent advances in understanding SCLC biology have identified Delta-like ligand 3 (DLL3) as a promising target for novel therapies. This review explores the evolving landscape of DLL3-targeted therapies in SCLC, examining their mechanistic basis, preclinical promise, and clinical development. We discuss various therapeutic modalities, including antibody-drug conjugates (ADCs), bispecific T-cell engagers (BiTEs), chimeric antigen receptor T-cell (CAR-T) therapies, and emerging approaches such as near-infrared photoimmunotherapy (NIR-PIT) and radiopharmaceutical therapy (RPT). The review highlights the challenges encountered in translating these promising approaches into clinical practice, including the setbacks faced by early DLL3-targeted therapies like Rovalpituzumab Tesirine (Rova-T). We also explore potential strategies to overcome these obstacles, emphasizing the need for a more nuanced understanding of DLL3 biology and its role in SCLC pathogenesis. The integration of cutting-edge technologies and interdisciplinary collaboration is proposed as a path forward to optimize DLL3-targeted therapies and improve outcomes for SCLC patients. This comprehensive overview provides insights into the current state and future directions of DLL3-targeted therapies, underscoring their potential to revolutionize SCLC treatment paradigms.

## 1 Introduction

Small cell lung cancer (SCLC) represents one of the most aggressive and lethal forms of lung malignancy, accounting for approximately 13-17% of all lung cancer cases (1–3). Characterized by rapid proliferation, early metastasis, and a high propensity for developing drug resistance, SCLC poses significant challenges in both diagnosis and treatment (4–6). The current standard of care for limited-stage SCLC typically involves a multimodal approach, combining platinum-based chemotherapy (usually cisplatin or carboplatin with etoposide) and concurrent radiotherapy. For patients achieving complete remission, prophylactic cranial irradiation is often recommended to mitigate the risk of brain metastases (7, 8). In extensive-stage SCLC, recent advancements have led to the incorporation of immunotherapy, specifically PD-L1 inhibitors, alongside traditional chemotherapy regimens (8, 9). However, the rapid development of drug resistance, frequent relapses, and significant treatment-related toxicities continue to hamper long-term survival outcomes (10). While immune checkpoint inhibitors (ICIs) have revolutionized treatment paradigms in various cancer types, their efficacy in SCLC has been limited. Although the high mutational burden of SCLC theoretically suggests potential responsiveness to immunotherapy, only a subset of patients derive significant benefit from the addition of ICIs to first-line chemotherapy (11, 12). Several factors contribute to the limited efficacy of ICIs in SCLC, including the downregulation of major histocompatibility complex (MHC) molecules, impaired antigen presentation, and substantial intratumoral heterogeneity (13, 14). Nevertheless, large-scale international studies have demonstrated improved survival outcomes when combining PD-L1 inhibitors, such as durvalumab, with chemotherapy, highlighting the potential of chemoimmunotherapy approaches in SCLC (15). Early analyses suggest that patients with inherently more immunogenic tumors may be the primary beneficiaries of such combination strategies (1, 8).

Given the limitations of current treatment modalities, there is an urgent need for novel therapeutic approaches in SCLC. Recent advances in molecular profiling and our understanding of SCLC biology have paved the way for the development of targeted therapies. These emerging strategies aim to address the complex mechanisms underlying SCLC progression and therapeutic resistance, potentially ushering in a new era of personalized medicine for this challenging malignancy (16, 17). One particularly promising avenue of research focuses on Delta-like ligand 3 (DLL3), a protein integral to the Notch signaling pathway that exhibits significant overexpression in specific SCLC subtypes, particularly those with pronounced neuroendocrine characteristics (18, 19). The unique expression pattern of DLL3, predominantly localized to SCLC cells with minimal presence in normal tissues, has positioned it as an attractive target for precision medicine strategies. Recent molecular profiling has revealed a strong correlation between DLL3 overexpression and the SCLC-A and SCLC-N subtypes, which are distinguished by elevated neuroendocrine gene expression (20).

Mechanistic studies have elucidated DLL3’s role in SCLC pathogenesis, demonstrating its involvement in cellular proliferation and metastatic processes (21, 22). Paradoxically, while high DLL3 expression appears to suppress Notch signaling in SCLC, reduced DLL3 levels may disrupt the typical lateral inhibition observed in Notch/DLL interactions, suggesting a complex regulatory mechanism (23).

The therapeutic landscape targeting DLL3 has seen both setbacks and promising developments. Rovalpituzumab tesirine (Rova-T), the pioneering antibody-drug conjugate aimed at DLL3, showed initial promise but was ultimately discontinued due to limited efficacy in phase 3 trials (24). However, this setback has not deterred further innovation in DLL3-targeted therapies. Emerging bispecific T-cell engagers (BiTEs), exemplified by tarlatamab (formerly AMG757), represent a new frontier in DLL3-directed immunotherapy. These novel agents have demonstrated encouraging results in both preclinical models and early-phase clinical trials, leveraging the selective expression of DLL3 in SCLC to enhance therapeutic specificity and efficacy (25, 26). Despite these advancements, significant challenges persist in the development of DLL3-targeted therapies, particularly in addressing treatment resistance and improving long-term outcomes. While approved agents such as anlotinib, veliparib, and talazoparib have shown efficacy in prolonging progression-free survival, their long-term therapeutic impact and safety profiles require further evaluation (25, 26).

The ongoing exploration of DLL3’s role in SCLC and the continuous refinement of targeted therapies underscore the dynamic nature of SCLC research. As our understanding of the molecular underpinnings of SCLC expands, so too does the potential for developing more effective, personalized treatment strategies (2, 27).

In this review, we will delve deeper into the evolving landscape of DLL3-targeted therapies in SCLC, examining their mechanistic basis, preclinical promise, and clinical development. We will discuss various therapeutic modalities, including antibody-drug conjugates, BiTEs, and chimeric antigen receptor T-cell therapies(CAR-T). Additionally, we will address the challenges encountered in translating these promising approaches into clinical practice and explore potential strategies to overcome these obstacles, with the ultimate goal of improving outcomes for patients facing this aggressive malignancy.

## 2 DLL3 in SCLC

### 2.1 Notch signaling pathway and DLL3

**Figure 1:**
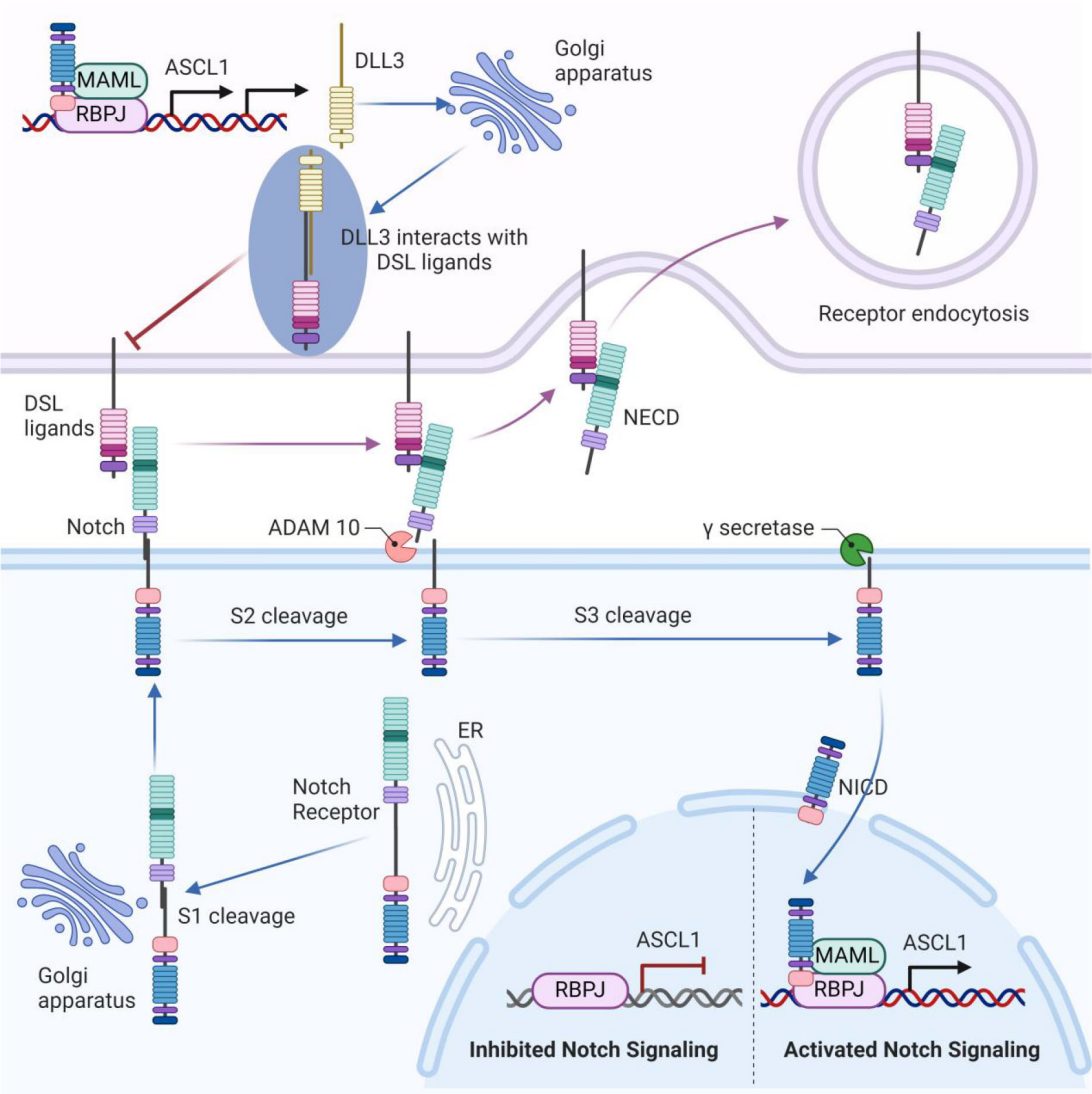
The unique role of DLL3 in the notch signaling pathway. The Notch receptor undergoes initial cleavage (S1) within the Golgi apparatus and is then transported to the cell membrane, where it interacts with DSL ligands on adjacent cells. Ligand binding triggers endocytosis and subsequent cleavages (S2 and S3), releasing the Notch intracellular domain (NICD), which translocates to the nucleus. In the nucleus, NICD forms a complex with RBPJ and MAML, regulating downstream target genes. This process inhibits ASCL1, which upregulates Notch ligand expression, including DLL3. DLL3 is primarily localized intracellularly within the Golgi apparatus. Through binding with other DSL ligands, DLL3 might inhibit Notch signaling.

Notch signaling is a multifaceted pathway involved in tumor cell regulation, including mitosis, proliferation, and differentiation (28). The key components of this pathway include four receptors (Notch1–4) and five ligands from the Delta/Serrate/Lag-2 (DSL) family (Delta-like1, 3, 4, and Jagged 1, 2), as well as the non-canonical ligand Delta-like 1 homolog (DLK1). The Notch receptor undergoes synthesis and initial cleavage by a furin-like convertase (S1 cleavage) within the Golgi apparatus. It is then transported to the cell membrane, where it interacts with DSL ligands on adjacent cells (29). Ligand ubiquitylation triggers clathrin-mediated endocytosis (CME), leading to the opening of the negative regulatory region (NRR) and subsequent S2 and S3 cleavages (30, 31). These steps release the Notch intracellular domain (NICD), which translocates to the nucleus. In the nucleus, NICD forms a complex with the recombination signal binding protein for immunoglobulin kappa J region (RBPJ) and mastermind-like transcriptional coactivator (MAML), thereby regulating downstream target genes (32). This process inhibits Achaete-scute homolog 1(ASCL1), a transcription factor pivotal in the neuroendocrine differentiation of SCLC, while ASCL1 itself upregulates Notch ligand expression, including DLL3 (21, 33) (**Figure 1**).

Unlike other DSL ligands that are present on the cell surface, DLL3 is primarily localized intracellularly within the Golgi apparatus (34). The function of DLL3 in Notch signaling remains a subject of debate. Some research suggests that DLL3 does not directly activate Notch signaling (35), whereas others indicate that DLL3 might inhibit Notch signaling by interacting with certain DSL ligands (36) (**Figure 1**). Ligand-receptor interactions in Notch signaling occur via trans-interaction (between cells) and cis-interaction (within the same cell), with DLL3 uniquely participating in cis-inhibition among the DSL ligands (35). Studies by Gavin et al. show that DLL3 co-localizes with Notch1, forming a complex that is targeted to lysosomes for degradation, thereby reducing Notch1 levels (37). In thymocytes, DLL3’s inhibition of Notch signaling enhances the positive selection process (38). Despite different views on the exact mechanism, DLL3 is recognized as an inhibitor of Notch signaling, making it a significant target for therapeutic strategies in cancers with aberrant Notch activity, such as SCLC.

### 2.2 DLL3 expression and its correlation with SCLC

**Figure 2:**
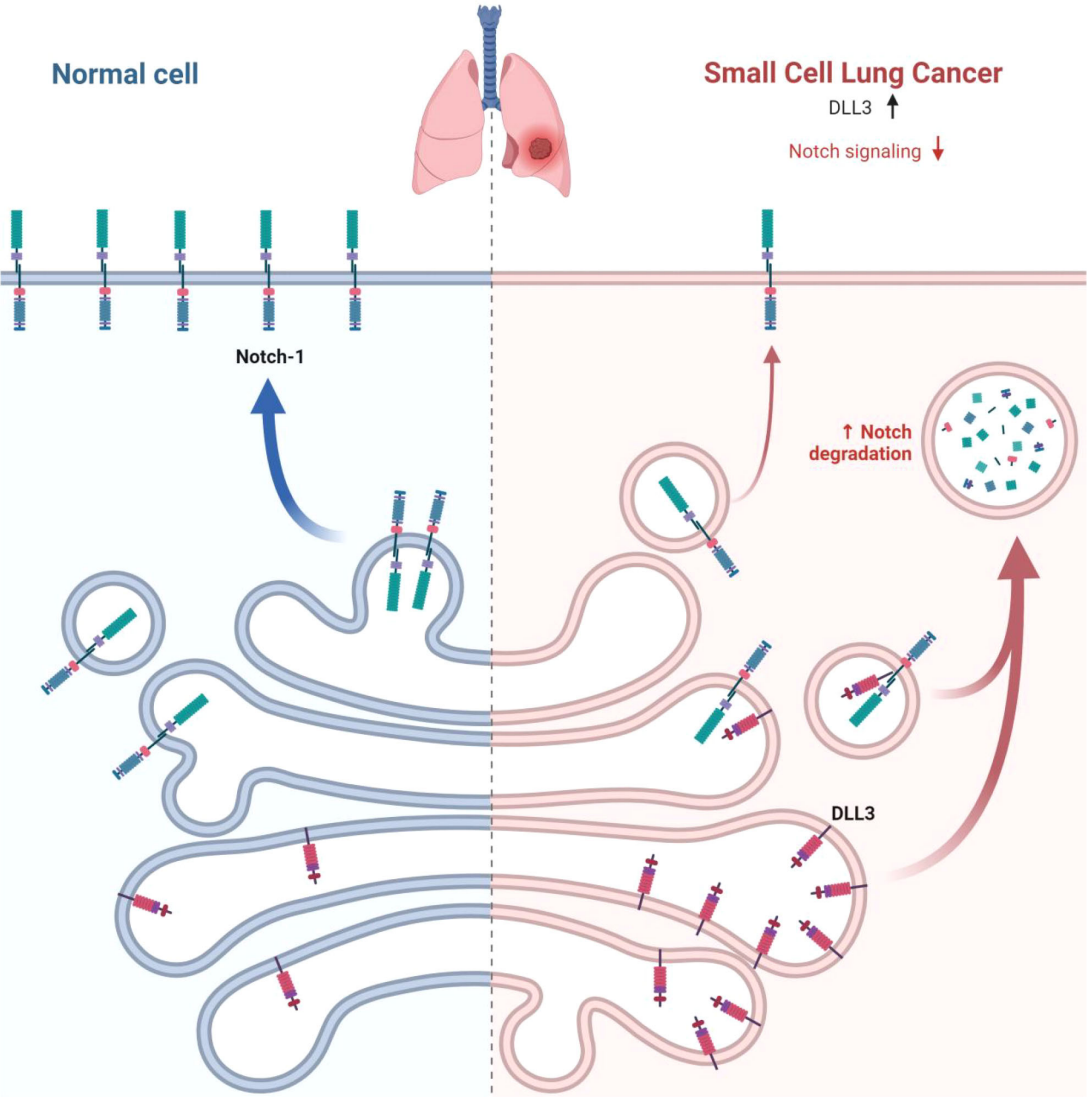
DLL3 overexpression in SCLC. In normal cells, Notch receptors bind to ligands on the surface of adjacent cells, leading to normal cellular processes. In SCLC cells, DLL3 is overexpressed, while Notch signaling is downregulated. This dysregulation of the Notch pathway contributes to the development and progression of SCLC.

The relationship between DLL3 and SCLC has been extensively investigated, revealing a complex interplay that, while not fully elucidated, offers intriguing insights into SCLC biology and potential therapeutic avenues (39–41). DLL3’s distinctive expression pattern - markedly elevated in SCLC tissues but virtually absent in normal tissues - suggests a pivotal role in the neuroendocrine characteristics that define SCLC (42)(**Figure 2**). This expression profile extends to large cell neuroendocrine carcinoma, hinting at a shared neuroendocrine etiology between these malignancies (43). The prognostic implications of DLL3 expression in SCLC have been a subject of considerable debate within the scientific community. Some researchers have reported a correlation between high DLL3 expression and poor clinical outcomes, including reduced overall survival (44, 45). However, these findings are not universally supported, with other studies failing to establish a significant link between DLL3 expression levels and patient survival or clinicopathological features (40, 41). Several factors may account for the discrepancies observed across various studies examining DLL3’s role in SCLC: (1) Genetic and environmental variations among different ethnic populations could influence DLL3 expression patterns and their clinical implications (46) (**Table 1**). (2) The nature of the tissue samples analyzed (biopsy versus surgical resection) may impact the assessment of DLL3 expression (47). (3) Statistical power and reliability of results can be affected by variations in sample sizes across different studies (46). (4) The predominant use of immunohistochemistry (IHC) for DLL3 detection introduces a degree of subjectivity that could contribute to inter-study variability. (5) Inconsistencies in defining thresholds for high DLL3 expression complicate the interpretation and comparison of results across studies.

**Table 1:** The expression of DLL3 in SCLC.

Country	Sample size	DLL3 positive rate	Survival	Reference
China	335	DLL3-low(38%) DLL3-high(62%)	DLL3-high (OS) < DLL3-low (OS)	(44)
China	38	100%	DLL3-high(MST:12months) DLL3-low (MST:23months)	(45)
China	247	DLL3-low(24%) DLL3-high(76%)	NS	(39)
Japan	63	83%	DLL3-high(OS:12.5months) DLL3-low(OS:15.7months)	(41)
Japan	95	83%	DLL3-high(OS:24.4months) DLL3-low (OS:33.3months)	(40)
America	44	79.5%	NS	(47)
Sweden	46	83%	NE	(48)
Germany	42	chemonaive 86.6% chemorelapsed 80%	NS	(49)

OS, overall survival; MST, median survival time; NE, Not evaluated; NS, No differences.

Despite these challenges, the scientific consensus largely supports a significant association between DLL3 expression and SCLC pathogenesis. The stark contrast in DLL3 expression between SCLC tissues and normal tissues underscores its potential as both a diagnostic biomarker and a therapeutic target. Future research directions should focus on standardizing DLL3 detection and quantification methods, conducting larger-scale studies with diverse patient populations, and exploring the molecular mechanisms underlying DLL3’s role in SCLC progression. Such efforts will be crucial in clarifying DLL3’s prognostic value and optimizing its utilization in targeted therapies for SCLC.

## 3 DLL3 target therapy in SCLC

The distinct overexpression of DLL3 in SCLC has catalyzed a new era in targeted therapy development for this aggressive malignancy (19). While predominantly localized within the Golgi apparatus, a subset of DLL3 molecules migrate to the cell surface, presenting an accessible target for therapeutic interventions (19). The negligible expression of DLL3 in healthy tissues confers a significant advantage, potentially minimizing off-target effects and enhancing the therapeutic index of DLL3-directed treatments (18). Current research efforts are focused on harnessing the Notch signaling pathway in SCLC through DLL3-targeted therapies. This approach has spawned a diverse array of therapeutic modalities, each employing unique mechanisms to exploit DLL3’s preferential expression on SCLC cells (50). These innovative strategies include: (1) Antibody-Drug Conjugates (ADCs): These biopharmaceuticals combine the specificity of monoclonal antibodies with potent cytotoxic agents, enabling precise delivery of cell-killing payloads to DLL3-expressing tumor cells. (2) BiTEs: These engineered proteins simultaneously bind to DLL3 on tumor cells and CD3 on T cells, facilitating targeted immune-mediated tumor cell destruction. (3) CAR-T Therapy: This approach involves genetically modifying a patient’s T cells to express receptors specific for DLL3, creating a personalized treatment that can recognize and eliminate SCLC cells.

As research progresses, the scientific community anticipates that DLL3-directed therapies will play an increasingly pivotal role in SCLC treatment strategies. The ongoing clinical trials are poised to provide critical insights into the optimal use of these innovative treatments, potentially revolutionizing the standard of care for SCLC patients. The clinical landscape for DLL3-targeted therapies in SCLC is rapidly evolving, with numerous trials underway to evaluate their safety profiles, efficacy, and optimal integration into treatment paradigms. These studies, summarized in comprehensive trial databases (**Table 2**), aim to elucidate the most effective applications of these novel therapies in improving patient outcomes.

**Table 2:** Ongoing clinical trials with new treatment strategies targeting DLL3.

Treatment		Patient number	Phase	Primary Endpoint	Trial ID
ADC	ZL-1310	140	I	Safety/MTD	NCT06179069
BiTE	tarlatamab	382	I	Safety/MTD	NCT03319940
		192	II	ORR	NCT05060016
		340	I	Safety/MTD	NCT05361395
		50	Ib	Safety/MTD	NCT04885998
		700	III	OS	NCT05740566
		222	III	OS	NCT06211036
		400	III	PFS	NCT06117774
	BI764532	110	I	Safety/MTD	NCT04429087
		30	I/II	Safety/MTD	NCT05879978
		120	I	ORR	NCT05882058
		44	I	Safety/MTD	NCT05990738
		60	I	Safety/MTD	NCT06077500
	QLS31904	290	I	Safety/MTD	NCT05461287
TiTE	HPN328	57	I/II	Safety/MTD	NCT04471727
	RO7616789	168	I	Safety/MTD	NCT05619744
CAR-T	AMG119	6	I	Safety/MTD	NCT03392064
	LB2102	41	I	Safety/MTD	NCT05680922
CAR-NK	NK-92	18	I	Safety/MTD	NCT05507593

ADC, Antibody-drug conjugate; BiTE, Bispecifc T-cell engager; CAR-NK, Chimeric antigen receptor nature killer cell therapy; CRT-T, Chimeric antigen receptor T-cell therapy; MTD, Maximal tolerable dosage; ORR, Objective response rate; OS, Overall survival; TiTE, Trispecifc T-cell engager; PFS, Median progression-free survival.

In conclusion, the development of DLL3-targeted therapies exemplifies the power of translating molecular insights into novel treatment modalities. As these approaches continue to be refined and evaluated in clinical settings, they hold the promise of significantly improving the therapeutic landscape for SCLC, a malignancy that has long challenged conventional treatment paradigms.

### 3.1 Rovalpituzumab tesirine

**Figure 3:**
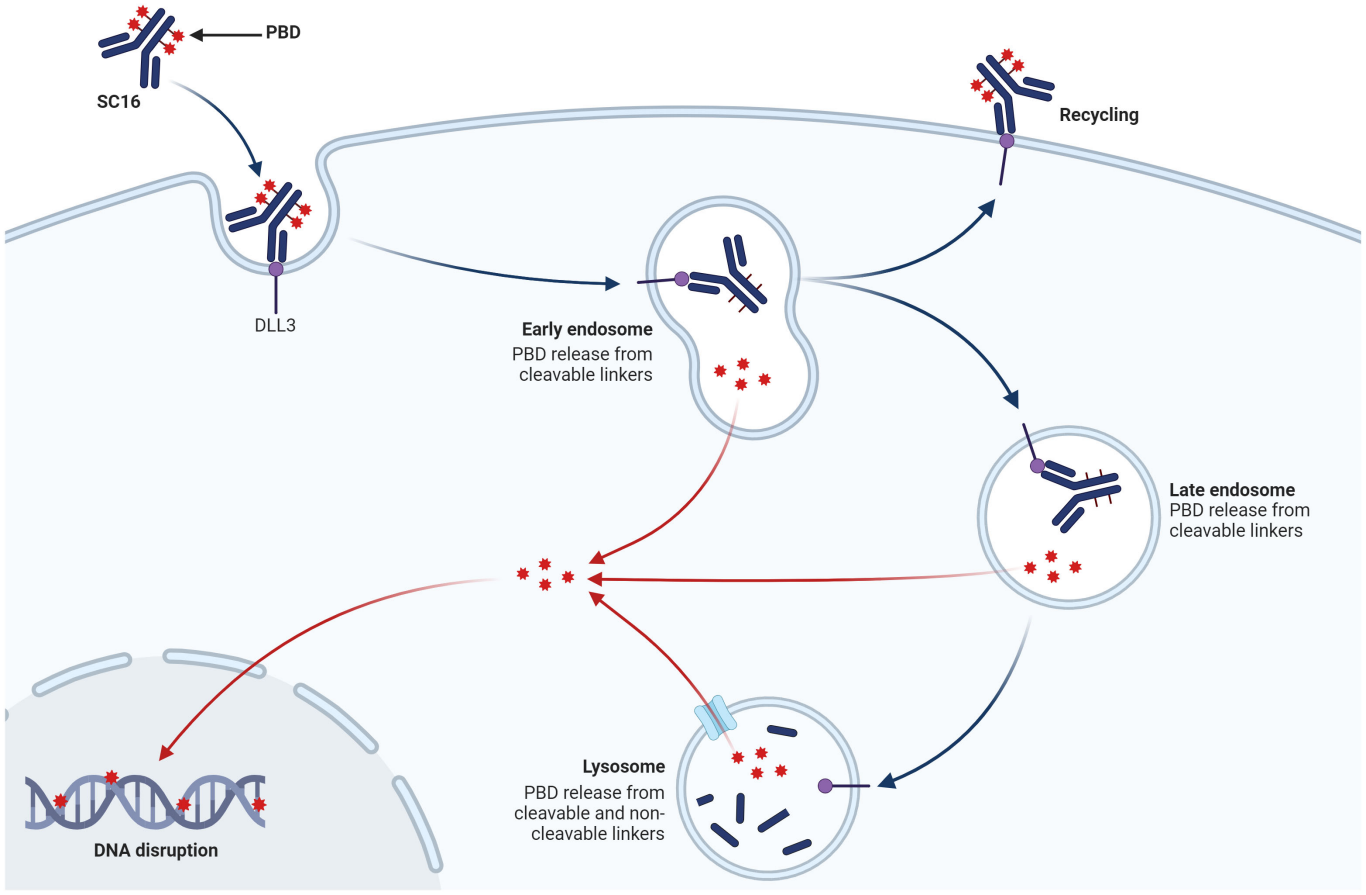
The mechanism of action of ADC targeting DLL3 on SCLC cells. Rova-T consists of a humanized anti-DLL3 monoclonal antibody (SC16) linked to a PBD dimer toxin payload via a cleavable linker. The ADC binds specifically to DLL3. As the ADC traffics to the lysosome, the linker is cleaved, releasing the PBD payload. The free PBD toxin then enters the nucleus, where it cross-links DNA, causing DNA disruption and ultimately leading to tumor cell apoptosis.

Rova-T’s sophisticated design incorporates a humanized anti-DLL3 antibody conjugated to a potent pyrrolobenzodiazepine (PBD) dimer via a cleavable linker. This architecture enables targeted payload delivery, theoretically amplifying antitumor effects while minimizing off-target toxicity (18, 51, 52) (**Figure 3**). Preclinical investigations utilizing patient-derived xenograft models demonstrated Rova-T’s robust efficacy, including activity against platinum-refractory SCLC variants. These encouraging results propelled the agent into clinical evaluation (18).

Initial human trials validated Rova-T’s safety profile and revealed promising efficacy signals, particularly in tumors with high DLL3 expression (51, 53). Subsequent studies explored synergistic potential with established therapies, yielding heterogeneous outcomes (54, 55). The pivotal phase II TRINITY trial further evaluated Rova-T in a cohort of heavily pretreated SCLC patients. While demonstrating activity, the results underscored the complexities of translating early-phase successes into broader clinical benefits (24). However, Rova-T encountered significant challenges in phase III trials. The MERU study, assessing its role in maintenance therapy, and the TAHOE trial, comparing it to standard second-line treatment, both failed to demonstrate superiority over existing approaches (56, 57). These disappointing outcomes precipitated the premature termination of both studies.

Throughout its development, Rova-T exhibited a multifaceted toxicity profile. Adverse events spanned hematological abnormalities, effusions, and dermatological reactions (24, 51, 57, 58). The underlying mechanisms of these toxicities, potentially linked to premature payload release or bystander effects, remain subjects of ongoing investigation (24, 57, 59, 60).

The Rova-T saga illuminates the intricate challenges inherent in developing targeted therapies for aggressive malignancies like SCLC. Despite its ultimate discontinuation, the experience has yielded invaluable insights into DLL3-targeted approaches and the broader landscape of precision oncology. Moving forward, researchers continue to explore alternative strategies for exploiting DLL3 in SCLC. Lessons gleaned from the Rova-T experience are informing the development of next-generation therapeutics, with emphasis on enhancing efficacy while mitigating toxicity. These efforts may encompass novel payload classes, advanced linker technologies, or alternative targeting modalities.

### 3.2 Bispecific T-cell engager molecules

BiTEs represent a pioneering immunotherapeutic strategy in oncology, harnessing the body’s immune defenses to combat malignancies. These innovative molecules are engineered to simultaneously bind T cells and tumor-specific antigens, effectively bridging the gap between immune effectors and cancer cells (61–63).

**Figure 4:**
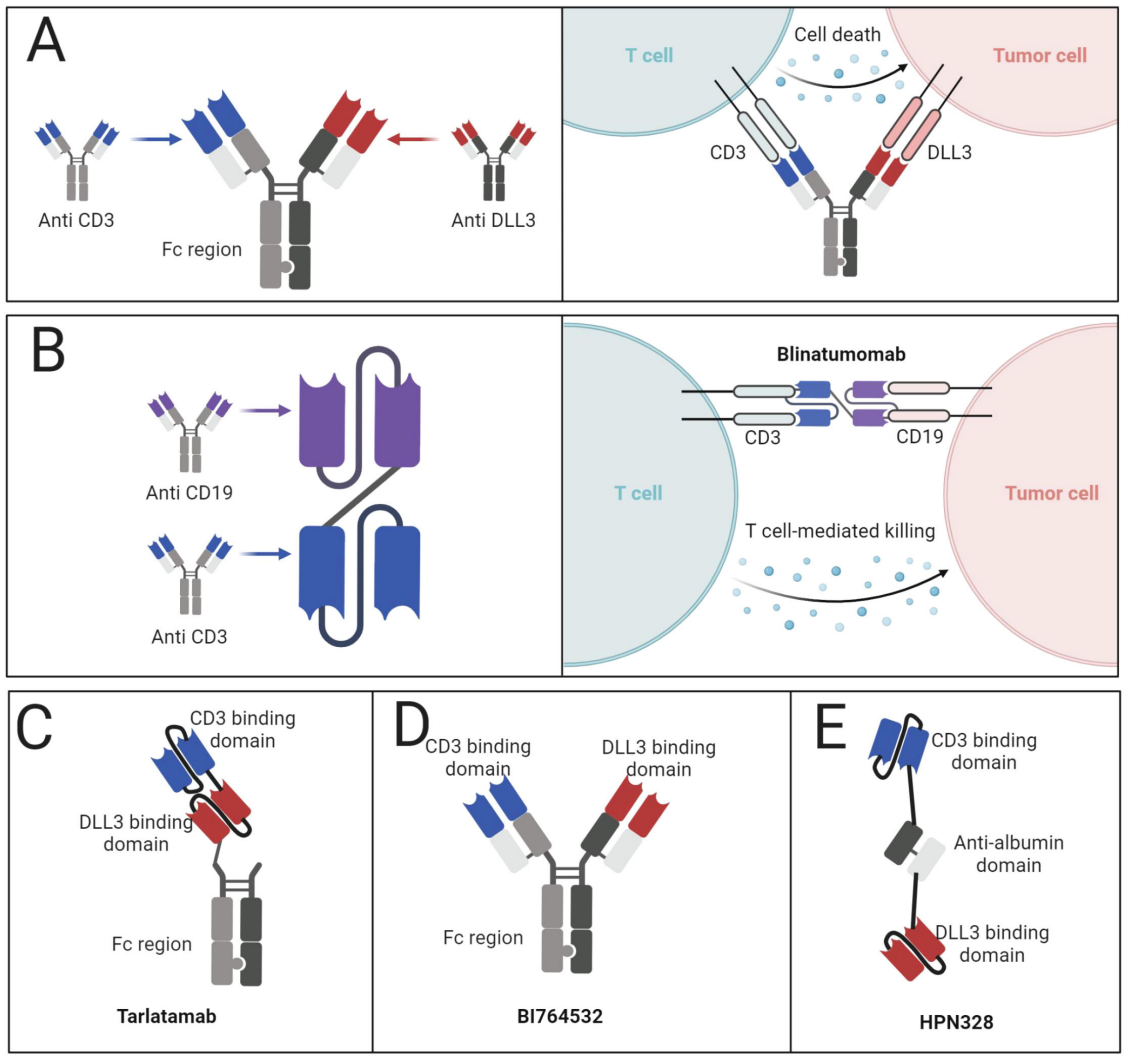
BiTEs targeting DLL3. **(A)** Mechanism of action of BiTEs targeting DLL3. **(B)** Structure of Blinatumomab and the mechanism of action. **(C)** Structure of tarlatamab. **(D)** Structure of BI764532. **(E)** Structure of HPN328.

The mechanism of action of BiTEs involves the formation of a highly specific immunological interface between T lymphocytes and neoplastic cells. This interaction triggers T-cell activation, proliferation, and the subsequent release of cytolytic granules and inflammatory mediators, culminating in tumor cell destruction (**Figure 4A**) (64–66). A notable advantage of BiTE technology lies in its ability to circumvent major histocompatibility complex class I (MHC-I) restrictions, rendering it particularly effective against tumors that have evolved to evade immune surveillance through MHC-I downregulation (67). The clinical trajectory of BiTEs has been marked by steady progress. Blinatumomab emerged as the vanguard, securing FDA approval for specific subtypes of acute lymphoblastic leukemia (**Figure 4B**). Subsequent approvals have expanded the BiTE landscape to include therapies for uveal melanoma, follicular lymphoma, and multiple myeloma, underscoring the versatility of this approach (61, 62). Tarlatamab (AMG757) (**Figure 4C**) has emerged as a promising DLL3-targeted BiTE. Preclinical investigations utilizing diverse tumor models demonstrated significant anti-neoplastic activity, including notable tumor regression and complete responses (68). The agent’s mechanism of action involves the promotion of T-cell infiltration, activation, and cytokine production within the tumor microenvironment. Clinical evaluation of tarlatamab in the DeLLphi-300 trial revealed encouraging efficacy in pretreated SCLC patients, with meaningful response rates and survival outcomes (69). The subsequent DeLLphi-301 study explored dose optimization, balancing efficacy with adverse event profiles (70). Tarlatamab’s development has progressed to phase III trials, with its biologics license application under regulatory review.

The BiTE landscape for SCLC extends beyond tarlatamab, encompassing molecules such as BI764532 (**Figure 4D**), HPN328 (**Figure 4E**), and QLS31904 (71, 72). These agents employ various structural modifications to enhance pharmacokinetics and efficacy. For instance, HPN328 incorporates albumin-binding properties to prolong half-life, while RO7616789 targets both DLL3 and CD137 to amplify T-cell responses (73). Early-phase clinical data for BI764532 and HPN328 have demonstrated promising anti-tumor activity with manageable toxicity profiles (20). These preliminary results underscore the potential of DLL3-targeted BiTEs in addressing the unmet needs of patients with advanced SCLC and other neuroendocrine malignancies.

The unique attributes of BiTE technology, including favorable pharmacokinetics and tolerable safety profiles, support continued investigation in this field. As clinical trials progress, these innovative therapies may reshape the treatment paradigm for historically refractory cancers, offering new avenues of hope for patients with limited therapeutic options.

### 3.3 Chimeric antigen receptor T cell therapy

**Figure 5:**
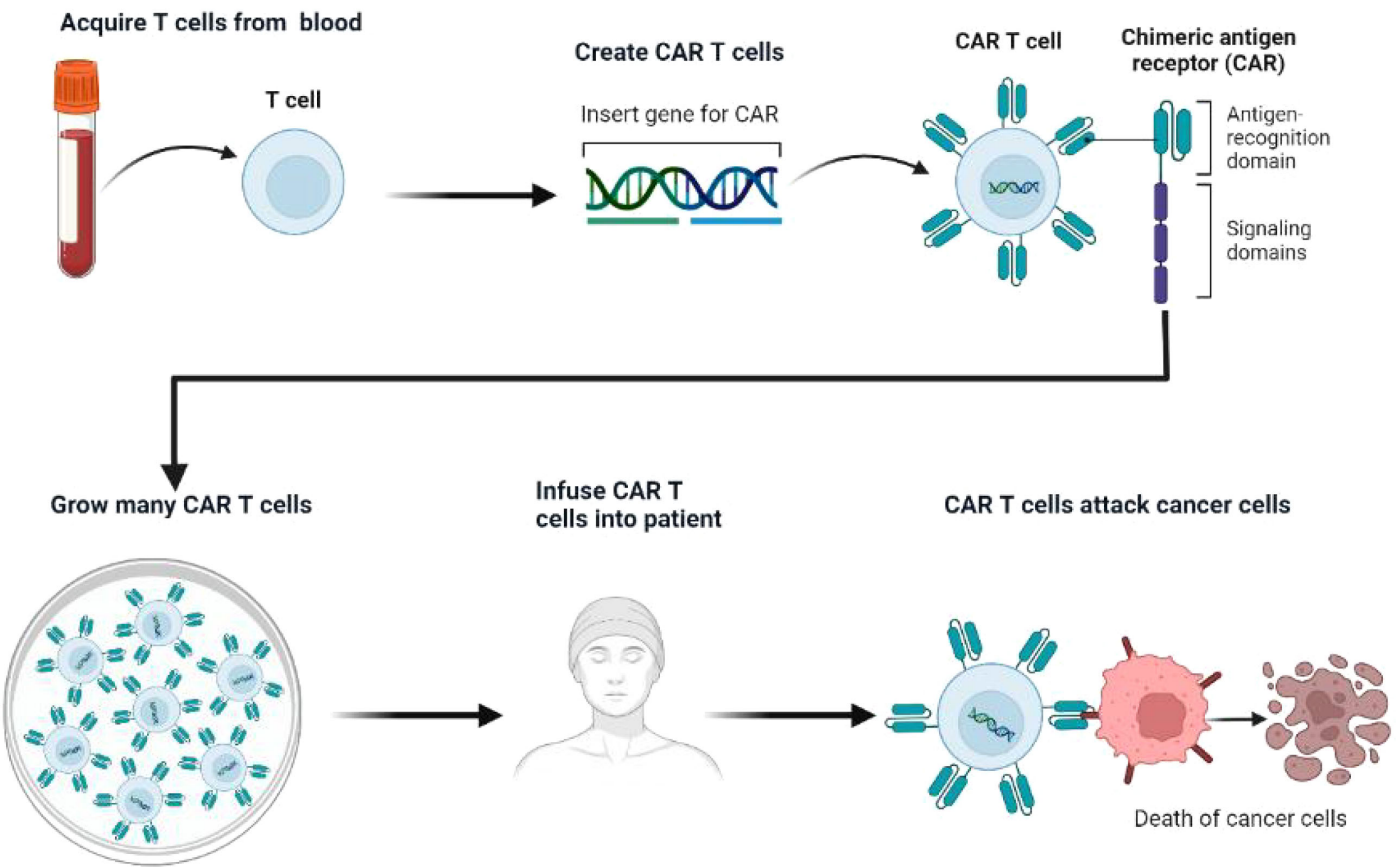
Diagram of CAR-T.

CAR T-cell therapy involves a sophisticated process of genetic engineering, wherein a patient’s T lymphocytes are modified to express synthetic receptors targeting specific tumor antigens(**Figure 5**) (74). In the context of SCLC, DLL3 serves as the primary target, enabling these engineered T cells to selectively recognize and eliminate DLL3-expressing neoplastic cells (75).

AMG 119 stands at the forefront of DLL3-targeted CAR-T therapies. This advanced cellular therapy incorporates a lentiviral vector encoding multiple functional domains: an anti-DLL3 binding moiety, co-stimulatory elements (CD28 and 4-1BB), and a CD3ζ signaling domain. Preclinical investigations have demonstrated AMG 119’s potent cytotoxicity against DLL3-positive SCLC cells and significant anti-tumor efficacy in xenograft models (76, 77). The clinical evaluation of AMG 119 commenced with a first-in-human phase I dose-escalation study (NCT03392064), enrolling patients with refractory or relapsed SCLC. This trial provided initial insights into the therapy’s safety profile and potential efficacy (78). The study revealed promising signs of clinical activity, including tumor regression in some patients, alongside a manageable safety profile. Importantly, the engineered T cells demonstrated robust *in vivo* expansion and persistence, key factors in the success of cellular therapies (79).

The landscape of DLL3-targeted cellular therapies extends beyond AMG 119, encompassing other CAR T-cell candidates such as LB2102 and ALLO-213, which are currently in preclinical stages of development. Furthermore, the field has expanded to include CAR-modified natural killer (NK) cells, offering an alternative cellular platform for targeting DLL3-positive tumors (79, 80). These NK-based therapies leverage the innate cytotoxic properties of NK cells, enhanced by CAR engineering to specifically target DLL3. Preclinical studies have demonstrated their potent and selective anti-tumor activity against DLL3-expressing cell lines and in animal models of pulmonary metastasis. The initiation of a phase I trial investigating DLL3-CAR-NK cells in extensive-stage SCLC (NCT05507593) underscores the growing interest in diverse CAR-based approaches for this challenging malignancy.

The advancement of DLL3-directed CAR therapies represents a significant leap in translating the success of cellular immunotherapies from hematological malignancies to solid tumors. The encouraging preclinical and early clinical data from AMG 119 and related therapies suggest that targeting DLL3 could provide a viable and potentially transformative treatment option for SCLC patients.

### 3.4 Chimeric antigen receptor NK cells for SCLC

Recent advancements in immunotherapy have led to the development of chimeric antigen receptor technology for NK cells, offering a promising approach for SCLC treatment. CAR-NK cells combine the innate cancer-fighting abilities of NK cells with the precision of CAR-mediated targeting. The structure of a CAR typically includes an extracellular antigen-recognition domain, a transmembrane region, and intracellular signaling components. For NK cells, the co-stimulatory domain CD137 (4-1BB) is often utilized to enhance activation (81, 82). CAR-NK cells can be derived from various sources, including primary adult NK cells, cord blood-derived NK cells, or established NK cell lines such as NK-92 (83). The use of NK-92 cells offers advantages in terms of standardization and availability as an “off-the-shelf” product.

While initial CAR-NK research focused on hematological malignancies, there is growing interest in their application for solid tumors, including SCLC. DLL3 has emerged as a promising target for SCLC due to its high expression in tumor tissues and limited presence in normal tissues (18). A recent study by Liu et al. demonstrated the effectiveness of DLL3-targeted CAR NK-92 cells against SCLC both *in vitro* and *in vivo* (80).

The advantages of CAR-NK cells over CAR-T cells include their ability to target tumors through both CAR-dependent and CAR-independent mechanisms, potentially reducing the risk of antigen escape. Additionally, CAR-NK cells have shown a lower risk of cytokine release syndrome and neurotoxicity (83).

Current research is focusing on enhancing the efficacy and persistence of CAR-NK cells in the tumor microenvironment. Strategies include incorporating genes for stimulatory cytokines like IL-15 (84), developing “armored” CARs resistant to immunosuppressive factors, and combining CAR-NK therapy with checkpoint inhibitors (85, 86). The study by Liu et al. demonstrated that DLL3-CAR NK-92 cells effectively targeted SCLC cells *in vitro* and induced tumor regression in both pulmonary metastasis and subcutaneous xenograft models. Importantly, the treatment showed a favorable safety profile with no significant side effects observed in treated mice (80).

While CAR-NK cell therapy for SCLC is still in preclinical stages, these early results suggest significant potential. As research progresses, CAR-NK cells may offer a valuable addition to the immunotherapy arsenal for SCLC, potentially providing a more accessible and safer alternative to CAR-T cell therapy. However, further clinical studies are needed to fully evaluate the safety and efficacy of this approach in SCLC patients.

### 3.5 Combination with immune checkpoint inhibitors

T cell-engaging therapies, exemplified by BiTEs, function by redirecting cytotoxic T lymphocytes to neoplastic cells, thereby enhancing immune infiltration within the tumor milieu. Concurrently, ICIs act to alleviate immunosuppressive pathways, reinvigorating exhausted T cells and promoting sustained anti-tumor responses. The rationale for combining these modalities stems from their potential to create a positive feedback loop, wherein each therapy potentiates the efficacy of the other. Preclinical investigations have elucidated the mechanistic underpinnings of this synergy. BiTE therapy has been observed to upregulate the expression of immune checkpoint molecules, including PD-1, PD-L1, and LAG-3, suggesting a biological basis for combining BiTEs with ICIs (71). Experimental models have corroborated this hypothesis, demonstrating enhanced anti-tumor efficacy when DLL3-targeted bispecific antibodies are administered in conjunction with PD-1 blockade (87).

The synergistic effects of this combination extend beyond SCLC, with studies in various solid tumor models revealing the potential to overcome resistance mechanisms and improve outcomes in tumors characterized by poor T cell infiltration (88). These findings underscore the broad applicability of this approach across different cancer types. However, the landscape of combinatorial immunotherapy in SCLC is not without complexity. While the integration of ICIs with BiTEs has shown promise, the combination of DLL3-targeting CAR-T cells with ICIs has yielded less consistent results in SCLC patients (87). Several hypotheses have been proposed to explain this discrepancy, including the potential inability of PD-1 inhibitors to prevent CAR-T cell depletion upon target engagement and possible detrimental effects on CAR-T cell functionality (89, 90). Interestingly, preclinical studies in murine SCLC models have demonstrated that the combination of AC133-specific CAR-T cells with both PD-1 blockade and CD73 inhibition resulted in enhanced tumor elimination and prolonged remission (91). This finding suggests that multi-modal approaches targeting diverse immunosuppressive mechanisms may hold the key to optimizing CAR-T cell efficacy in SCLC.

In recent research, combining DLL3-targeted therapies with ICIs has demonstrated notable efficacy in addressing SCLC’s characteristic immune evasion, often seen as a “cold” tumor environment. DLL3-targeted BiTEs, such as tarlatamab, engage DLL3-positive tumor cells and simultaneously attract T cells to these sites, promoting tumor infiltration. BiTE therapy has been shown to upregulate immune checkpoint molecules in the tumor microenvironment, providing a mechanistic basis for further combination with ICIs (87, 88). This combination can sustain T cell activity, reduce exhaustion, and potentiate anti-tumor responses over extended treatment periods. Similarly, DLL3-targeted CAR-T cells, such as AMG 119, show potential in combination with ICIs by addressing T cell exhaustion. ICIs can alleviate inhibitory signals in the tumor microenvironment, prolonging CAR-T cell persistence and efficacy against DLL3-expressing SCLC cells (78). Moreover, integrating other immunomodulatory agents, like CD73 inhibitors, may further enhance the efficacy of DLL3-targeted therapies by mitigating immune suppression in the SCLC microenvironment. Preclinical evidence indicates that CD73 inhibition enhances CAR-T cell infiltration and activity, highlighting the potential for multi-modal immunotherapy to transform “cold” tumors into “hot” ones with robust immune responses (91).

In summary, DLL3-targeted therapies combined with ICIs represent an innovative and promising approach to treating SCLC, offering both direct tumor targeting and immune modulation to achieve sustained anti-tumor responses. As research progresses, optimizing these combination strategies through a deeper understanding of the underlying mechanisms may lead to improved clinical outcomes and set new standards for managing this aggressive malignancy.

### 3.6 Near-infrared photoimmunotherapy

NIR-PIT represents a cutting-edge therapeutic modality that synergizes principles of photophysics and immunology to combat malignancies (92). This innovative approach employs a bespoke antibody-photoabsorber conjugate (APC) comprising a tumor-specific monoclonal antibody linked to IRDye700DX (IR700), a photosensitizing agent (93, 94). The mechanistic underpinnings of NIR-PIT involve a two-step process. Initially, the APC selectively accumulates on neoplastic cells expressing the target antigen. Subsequently, exposure to near-infrared light at 690 nm wavelength induces conformational changes in the IR700 moiety, precipitating localized membrane disruption and cellular demise (95). A distinguishing feature of NIR-PIT lies in its ability to induce immunogenic cell death (ICD). This process liberates an array of damage-associated molecular patterns (DAMPs), including calreticulin, heat shock proteins, ATP, and HMGB1. These molecular signals orchestrate a cascade of immunological events, culminating in the activation of dendritic cells and the subsequent priming of cytotoxic T lymphocytes against residual tumor antigens (96).

In the context of SCLC, DLL3 has emerged as a compelling target for NIR-PIT. The favorable tissue penetration characteristics of near-infrared light in pulmonary parenchyma enhance the feasibility of this approach for intrathoracic malignancies (97). Iisobe and colleagues have pioneered the development of a DLL3-targeted NIR-PIT platform. Their preclinical investigations, utilizing an anti-DLL3 monoclonal antibody conjugated to IR700, demonstrated significant tumor growth suppression and survival benefits in murine models of SCLC. Notably, this therapeutic approach exhibited a favorable safety profile with minimal off-target effects (98).

The dual mechanism of action inherent to NIR-PIT - direct tumor cell ablation coupled with the induction of systemic anti-tumor immunity - positions this modality as a promising frontier in SCLC therapeutics. By leveraging the tumor-specific expression of DLL3 and the unique properties of photoactivated cytotoxicity, DLL3-targeted NIR-PIT offers the potential for precise and minimally invasive intervention in SCLC.

As research in this field progresses, several avenues warrant exploration. Optimization of light delivery systems for intrathoracic applications, elucidation of optimal dosing and fractionation schedules, and investigation of potential synergies with existing immunotherapeutic approaches represent critical areas for future study. Furthermore, the integration of NIR-PIT into multimodal treatment paradigms for SCLC merits consideration. Its potential to induce ICD may enhance the efficacy of subsequent immunotherapies or chemotherapeutic regimens, potentially leading to more durable responses and improved patient outcomes.

### 3.7 Radiopharmaceutical therapy

RIT has emerged as a promising modality in oncology, synergizing the precision of targeted antibody delivery with the cytotoxic potential of radioisotopes. The success of 90Y-ibritumomab tiuxetan in non-Hodgkin’s lymphoma has paved the way for exploring similar approaches in other malignancies, including SCLC (99). In the context of SCLC, a novel radioimmunotherapeutic agent, [177Lu]Lu-DTPA-CHX-A”-SC16, has been developed by Tully and colleagues. This compound ingeniously combines the tumor-targeting capabilities of an anti-DLL3 monoclonal antibody (SC16) with the therapeutic β-emissions of Lutetium-177. This design allows for selective irradiation of DLL3-expressing SCLC cells while mitigating collateral damage to healthy tissues. Preclinical evaluations of [177Lu]Lu-DTPA-CHX-A”-SC16 have yielded encouraging results. In xenograft models utilizing the human SCLC cell line NCI-H82, the radiolabeled antibody demonstrated superior tumor control and survival benefits compared to its non-radioactive counterpart. These findings were further corroborated in patient-derived xenograft models, where dose-dependent antitumor activity was observed with manageable and transient toxicities, primarily affecting hematopoietic and hepatic systems. The persistent expression of DLL3 in residual tumor cells post-treatment suggests the potential for fractionated or repeated administrations of [177Lu]Lu-DTPA-CHX-A”-SC16 to enhance therapeutic efficacy. Moreover, the integration of this radioimmunotherapeutic approach with other DLL3-targeted modalities, such as bispecific T-cell engagers or chimeric antigen receptor T-cell therapies, presents an intriguing avenue for multimodal treatment strategies in SCLC. The immunomodulatory effects of radiation on the tumor microenvironment, particularly its impact on myeloid cell populations, provide a rationale for combining [177Lu]Lu-DTPA-CHX-A”-SC16 with immune checkpoint inhibitors. This combinatorial approach could potentially leverage radiation-induced immune activation to augment the efficacy of immunotherapy in SCLC. As [177Lu]Lu-DTPA-CHX-A”-SC16 progresses towards clinical evaluation, several key areas warrant investigation. Optimization of dosing regimens, elucidation of potential resistance mechanisms, and exploration of synergistic combinations with existing therapies will be crucial in maximizing its therapeutic potential. Furthermore, the development of companion diagnostics utilizing positron-emitting isotopes (e.g., 68Ga or 89Zr) conjugated to the anti-DLL3 antibody could enable non-invasive assessment of DLL3 expression and biodistribution, facilitating patient selection and treatment monitoring. In conclusion, the advent of [177Lu]Lu-DTPA-CHX-A”-SC16 represents a significant advancement in targeted radiotherapy for SCLC. By harnessing the tumor-specific expression of DLL3 and the therapeutic potential of beta-emitting radioisotopes, this approach offers a novel strategy for precision medicine in SCLC.

### 3.8 Patient selection and biomarker use in DLL3-targeted therapies

In DLL3-targeted therapies, optimizing patient selection and biomarker use is essential for enhancing therapeutic efficacy in SCLC. Due to the heterogeneity of DLL3 expression among SCLC patients, stratifying individuals based on DLL3 positivity has emerged as a key approach to maximizing treatment benefits. Studies in recent years demonstrate that patients with high DLL3 expression show greater responsiveness to targeted therapies. Both therapies are under clinical evaluation with patient cohorts specifically selected for high DLL3 expression, which serves as a critical biomarker for these targeted approaches (12, 69).

Moreover, combining DLL3 with other biomarkers, such as ASCL1 and PD-L1, has demonstrated further improvements in predicting patient response. ASCL1, a transcription factor regulating neuroendocrine differentiation, is frequently co-expressed with DLL3 in SCLC subtypes, especially those with pronounced neuroendocrine features. This co-expression offers a more refined selection strategy, allowing for a targeted approach that aligns with the unique genetic and molecular profiles of SCLC patients (33, 40). Additionally, PD-L1, another key biomarker in immunotherapy, has shown potential when used alongside DLL3. Studies suggest that patients with concurrent high expression of both DLL3 and PD-L1 may benefit more from combination therapies that pair DLL3-targeted agents with immune checkpoint inhibitors. This approach capitalizes on the synergistic potential between DLL3-targeted T-cell engagers and immunotherapy agents, especially in patients with immune-responsive SCLC tumors (71).

In parallel, advancements in diagnostic technologies are revolutionizing DLL3 detection and monitoring. Liquid biopsies, imaging techniques, and quantitative immunohistochemistry are enabling non-invasive, real-time assessment of DLL3 expression levels, offering a dynamic view of tumor biology during treatment. These techniques allow for more precise patient selection at baseline and facilitate continuous monitoring of DLL3 status, which is especially valuable in assessing treatment response and adapting therapeutic strategies accordingly (98). The recent development of standardized immunohistochemical methods further enhances the reproducibility and reliability of DLL3 detection across diverse clinical settings, thereby minimizing inter-laboratory variability and establishing DLL3 as a consistent biomarker for SCLC patient selection (47).

Together, these advances in patient selection and biomarker applications are paving the way for more personalized, effective DLL3-targeted therapies. As research progresses, refining these selection criteria and integrating multi-biomarker strategies will be crucial in optimizing therapeutic outcomes for SCLC patients and expanding the potential of DLL3-directed treatments.

## 4 The future of DLL3-targeted therapies in SCLC: A paradigm shift in precision oncology

The advent of DLL3-targeted therapies marks a watershed moment in the field of SCLC treatment, heralding a new epoch in precision oncology. This innovative approach holds the promise of revolutionizing SCLC management, offering hope to patients facing this aggressive malignancy. However, the journey from scientific discovery to clinical efficacy is fraught with complexities. The setbacks encountered in late-stage clinical trials of early DLL3-targeted therapies underscore the necessity for a more nuanced understanding of DLL3 biology within the intricate tapestry of SCLC pathogenesis. This calls for a paradigm shift in our conceptualization of DLL3, moving beyond its simplistic role as a binary biomarker towards a more sophisticated, dynamic model of its expression and function.

The future of DLL3-targeted therapies lies in the convergence of cutting-edge technologies and interdisciplinary collaboration. Next-generation sequencing platforms, coupled with advanced bioinformatics and machine learning algorithms, offer unprecedented opportunities to decipher the complexities of DLL3 expression at a systems level. By integrating multi-modal data streams - including genomics, transcriptomics, proteomics, and advanced imaging - we can construct a holistic model of DLL3 biology that accounts for spatial and temporal heterogeneity, as well as the complex interplay between tumor cells and their microenvironment.

An emerging and promising frontier in DLL3-targeted therapies is the development of induced pluripotent stem cell (iPSC)-derived immune cells. This innovative approach holds significant potential for enhancing the efficacy and scalability of cellular immunotherapies for SCLC. iPSC-derived T cells, NK cells, and other immune effectors can be engineered to target DLL3, potentially offering several advantages over traditional autologous cell therapies:

(1) Standardization: iPSC-derived immune cells provide a more consistent and scalable source of therapeutic cells, potentially reducing variability in treatment outcomes. (2) Enhanced functionality: These cells can be genetically modified more extensively than primary cells, potentially incorporating multiple targeting moieties or enhanced resistance to the immunosuppressive tumor microenvironment. (3) “Off-the-shelf” availability: iPSC-derived therapies could overcome the logistical challenges and production delays associated with autologous cell therapies, making treatment more readily available to patients. (4) Reduced cost: Large-scale production of iPSC-derived immune cells could potentially lower the cost of cellular immunotherapies, improving accessibility. Future research directions in this area should focus on optimizing the differentiation and expansion protocols for iPSC-derived immune cells, enhancing their *in vivo* persistence and anti-tumor efficacy, and developing strategies to mitigate potential safety concerns such as off-target effects or uncontrolled proliferation.

This comprehensive understanding will inform the development of novel therapeutic modalities that harness the specificity of DLL3 targeting while engaging the body’s innate immune defenses. Bifunctional molecules, such as BiTEs and CAR-T-cells, represent promising avenues for exploration. However, realizing their full potential will require a deep understanding of the immunological landscape of SCLC and innovative strategies to overcome the immunosuppressive barriers within the tumor milieu.The evolution of DLL3-targeted therapies necessitates a reimagining of the clinical trial paradigm. Traditional approaches may prove inadequate in the era of precision medicine, where therapies are tailored to increasingly specific molecular profiles. Adaptive trial designs, such as basket and umbrella trials, offer more agile frameworks for evaluating targeted therapies, allowing for real-time integration of biomarker data and rapid adjustment of treatment strategies.

Ultimately, the success of DLL3-targeted therapies in SCLC hinges on fostering a culture of open collaboration and interdisciplinary dialogue. By breaking down silos between academia, industry, and clinical practice, we can accelerate the pace of innovation and translate scientific discoveries into meaningful clinical outcomes.

## 5 Conclusion

DLL3-targeted therapies represent a promising frontier in SCLC treatment, offering new hope for patients facing this aggressive malignancy. While early setbacks with therapies like Rova-T highlight the complexities of targeting DLL3, they also underscore the need for a more nuanced understanding of DLL3 biology in SCLC. The future of these therapies lies in integrating cutting-edge technologies, such as advanced sequencing and bioinformatics, to construct a comprehensive model of DLL3 expression and function. Emerging approaches, including BiTEs, CAR-T cells, NIR-PIT, and RPT, show potential for overcoming current limitations. However, their success hinges on addressing challenges in the tumor microenvironment and optimizing delivery strategies. Reimagining clinical trial designs and fostering interdisciplinary collaboration will be crucial in translating these scientific advances into improved patient outcomes. As research progresses, DLL3-targeted therapies have the potential to transform the treatment landscape for SCLC, offering a more personalized and effective approach to combat this challenging disease.
